# Trichosporon Endophthalmitis Following Cataract Surgery: A Case Report

**DOI:** 10.7759/cureus.34067

**Published:** 2023-01-22

**Authors:** Saritrasaraswathy Thilagaraj, Mimiwati Zahari, Krishnan Sarojini, Fazilawati A Qamarruddin

**Affiliations:** 1 Ophthalmology, University Malaya, Kuala Lumpur, MYS; 2 Ophthalmology, Hospital Tengku Ampuan Rahimah, Klang, MYS

**Keywords:** voriconazole, phacoemulsification cataract surgery, fungal, type-2 diabetes mellitus, trichosporon species, normal ocular flora, complication, exogenous endophthalmitis

## Abstract

Fungal infections always pose a predicament to management and prognosis. The saprophytic fungus, *Trichosporon inkin* commonly causes endogenous infection in immunocompromised individuals. We report a case of exogenous *T. inkin* endophthalmitis successfully treated with voriconazole, pars plana vitrectomy, and removal of the source of infection. A 51-year-old gentleman with suboptimal control of diabetes presented with a right painful red eye for a week after undergoing an uneventful phacoemulsification with a posterior chamber intraocular lens implant more than a month prior. He presented with intense inflammation in the right anterior chamber that did not respond to steroid challenge. Ultrasound B scan showed vitreous opacities with no loculations. The culture of vitreous humor was negative. Systemic investigations were also normal. Despite being given multiple intravitreal antibiotics, his right eye got worse. He then underwent vitrectomy and intraocular lens explantation, in which the culture of the lens grew *Trichosporon Inkin*. He was subsequently started on the appropriate antifungals (topical, intravitreal, and systemic) based on the minimum inhibitory concentration of the antifungal sensitivity test. The patient eventually showed significant clinical improvement, and intraocular inflammation was subsiding after six months of treatment. His best corrected visual acuity improved to 6/12 with Snellen’s visual acuity chart.

## Introduction

*Trichosporon *(genus) organisms are a group of fungi that belong to the basidiomycetes division. They are ubiquitous but commonly found in areas that are warm and humid. In humans, they are part of the normal gastrointestinal and oral cavity flora and can transiently colonize the respiratory tract, skin, and genitalia [1].

*Trichosporon* usually causes superficial infection but can also cause invasive systemic fungemia, especially in immunocompromised patients [2]. *Trichosporon* species reported causing human infections are *T. asahii,* *T. cutaneum,* and *T. beigelii*, more commonly the former [3].

We are reporting a case of postoperative endophthalmitis caused by *Trichosporon inkin* without any systemic impact.

## Case presentation

A 51-year-old man with a history of suboptimal control of diabetes mellitus presented with a right painful red eye with blurred vision for almost a week. He had uneventful phacoemulsification and intraocular implantation six weeks ago. He had no history of ocular trauma or contact with vegetative matter or animals. He also has no high-risk behavior and did not have any constitutional symptoms. His best corrected visual acuity was 6/12 over the right eye using Snellen’s chart with meibomitis. The right eyelid was not swollen, and there was no chemosis. Slit-lamp biomicroscopy revealed significant inflammation in the anterior segment consisting of fine keratic precipitates and anterior chamber cells (3+) with no fibrin. The intraocular pressure measured using Goldmann applanation tonometry was 14 mmHg in the right eye. The intraocular lens was stable with no retrolental opacity. There were dense vitritis that masked the fundus view. Ultrasound B-scan showed vitreous opacities with no loculations, flat retina, and normal posterior scleral thickness. The left eye showed unremarkable ocular findings. Systemic examination was unremarkable as well.

**Figure 1:**
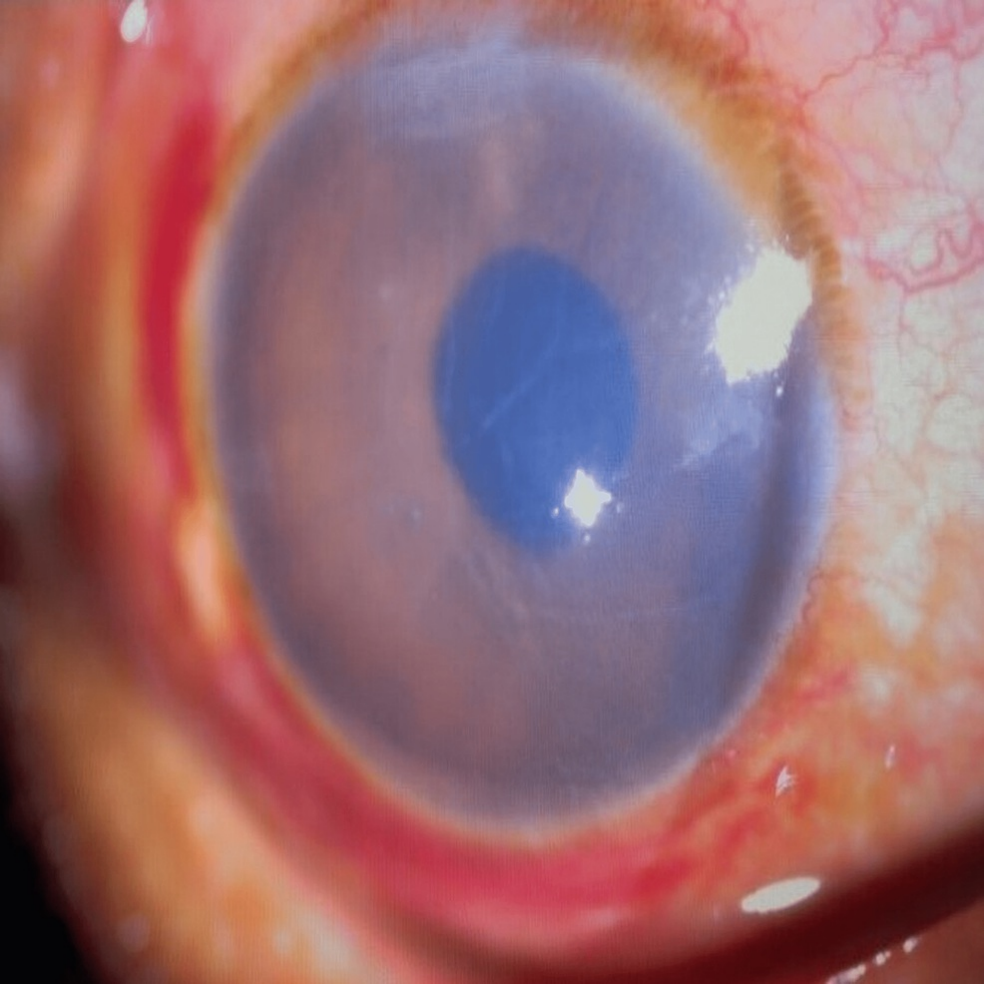
RE worsened with the more edematous cornea

The initial impression was right severe anterior uveitis, and a topical steroid challenge for two hours was given to the patient. His right eye subsequently worsened with the appearance of hypopyon (Figure 1). 

**Figure 2:**
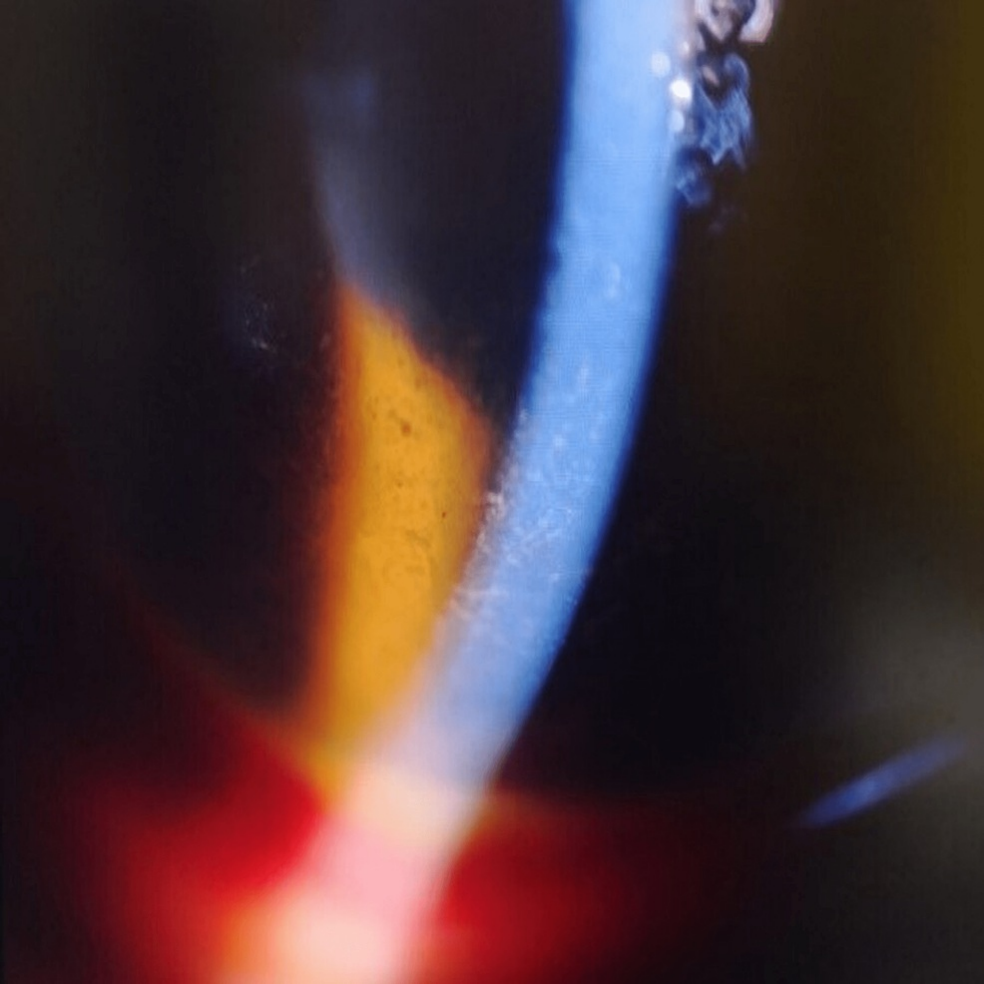
RE endothelial dusting and increased hypopyon despite multiple intravitreal antibiotics

**Figure 3:**
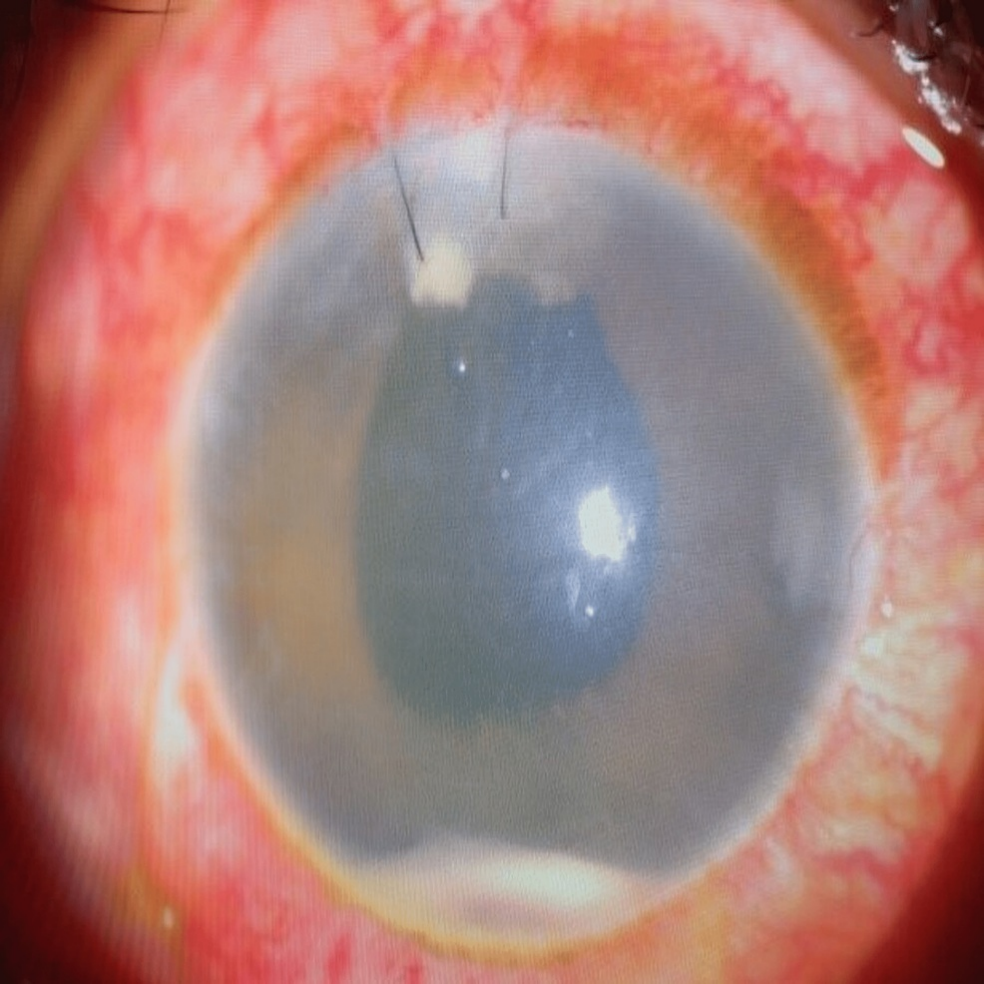
Reducing ocular inflammation (less hypopyon and corneal edema) a week after intraocular lens removal and commencement of Voriconazole

A diagnosis of right eye postoperative endophthalmitis was then made, and he was given intravitreal injections of vancomycin (1.0 mg/0.1 mL) and ceftazidime (1.0 mg/0.1 mL) following a vitreous tap. The patient was started on topical fortified vancomycin (50 mg/mL), ceftazidime (100 mg/mL), dexamethasone every hour, and intravenous ciprofloxacin 400mg twice daily. Vitreous humor culture was negative. He was given multiple intravitreal vancomycin (1.0 mg/0.1 ml) and ceftazidime (1.0 mg/0.1 ml). Unfortunately, his right vision further dropped with endothelial dusting and hemorrhagic hypopyon (Figures 2, 3).

Thus, further investigations were done to rule out any systemic infection. Blood investigations were normal. Cultures of blood, urine, and toenail were also negative. Chest X-ray showed clear lung fields, and ultrasound of the abdomen showed no intra-abdominal collection. 

He then underwent pars plana vitrectomy and intraocular lens extraction, in which culture of the lens grew *Trichosporon inkin*. He was started on antifungals, which included topical amphotericin B 0.15% and fluconazole 0.2%, and also given intravitreal amphotericin B (5 mg /0.1 mL). However, the inflammation worsened.

For antifungal sensitivity, the minimum inhibitory concentrations (MIC) that were determined using the reference broth microdilution method for fluconazole, voriconazole, amphotericin B, and caspofungin against the organism were 0.75 mg/L, 0.023 mg/L, 3 mg/L, and 32 mg/L, respectively, demonstrating that the fungus was resistant to amphotericin B but sensitive to voriconazole. Hence, topical antifungal treatments were switched from amphotericin B to voriconazole. Besides topical voriconazole (1mg/ml), systemic form (intravenous (4mg/kg for 2 weeks followed by oral 200mg BD) and together with multiple courses of intravitreal voriconazole (25 mg/0.1 mL) were also given.

**Figure 4:**
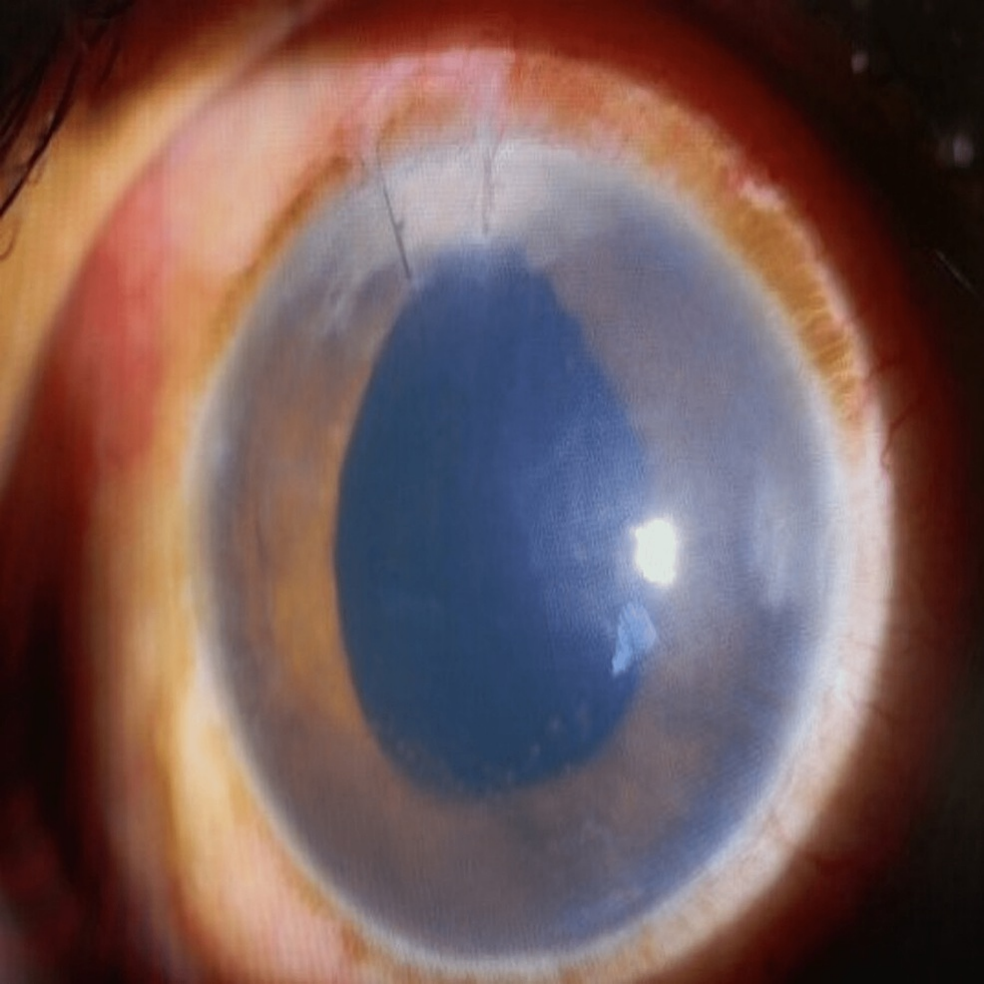
Resolution of ocular signs after completing voriconazole

This therapy commenced after liaising with the infectious disease physician. It eventually resulted in a significant clinical improvement and reduced intraocular inflammation after six months of treatment. His best corrected visual acuity improved to 6/12 with Snellen’s visual acuity chart (Figure 4).

## Discussion

Postoperative fungal endophthalmitis accounts for less than 10% of the microbial spectrum seen after cataract removal, which makes it relatively rare. Among the fungi species, *Candida, Aspergillus, and Fusarium spp* are the common ones that can lead to this blinding disease [4].

Postoperative endophthalmitis is categorized as chronic if the occurrence is after six weeks of surgery, such as in this case. It may have an early or late onset ranging from 48 hours to 7 months [4].

There are reports of exogenous endophthalmitis caused by *Trichosporon asahii* and *cutaneoum* with positive cultures acquired from the intraocular lens and structures of the enucleated eye, respectively [3,5]. As stated earlier, this species of fungi is a commensal of the skin, including the hair follicles. The most likely source of our patient’s ocular infection was his own local microflora which probably occurred during intraocular inoculation amid perioperative cataract surgery. The toenail culture of our patient was taken because he had onychomycosis. Although the culture turned out negative, this could be a probable source of infection in our patient. 

Furthermore, poorly controlled diabetes mellitus likely predisposed our patient to this infection, similarly as reported by Saban Gonul et al. in their publication [5].

The ability of this organism to adhere and form biofilms on implanted devices, such as intraocular lenses, can contribute to the progression of Trichosporonosis, as it aids in its escape from antifungals and host immune responses [6].

Another virulence factor of *Trichosporon* is that it expresses glucuronoxylomannan (GXM) in its cell wall, similar to *Cryptococcus neoformans*. GXM is a 1,3-linked mannan backbone attached to short side chains of 1,4-linked mannose and 1,2-linked xylose residues by substituting the 2 or 4 portions of the 1,3-linked mannose residues of the main group. This polysaccharide attenuates the phagocytic capability of neutrophils and monocytes within the human body [6].

Voriconazole, a wide spectrum triazole compared to other Azole drugs, is the best to treat* Trichosporon* based on the study done by Gulsen Hazirolan et al., proven by the lowest minimum inhibitory concentration (MIC) and highest killing rate, followed by itraconazole, posaconazole, isavuconazole and fluconazole [7].

## Conclusions

Fungal etiology should be strongly suspected in cases of chronic postoperative endophthalmitis, especially in the presence of debilitating comorbidities and poor response to standard treatment. *Trichosporon sp*. is one of the many fungi that can cause this blinding disease. Following the removal of the nidus of infection and with the help of minimum inhibitory concentration, it can be successfully treated with the right antifungal.
